# Correlation of *KIT* and *PDGFRA* mutational status with clinical benefit in patients with gastrointestinal stromal tumor treated with sunitinib in a worldwide treatment-use trial

**DOI:** 10.1186/s12885-016-2051-5

**Published:** 2016-01-15

**Authors:** Peter Reichardt, George D. Demetri, Hans Gelderblom, Piotr Rutkowski, Seock-Ah Im, Sudeep Gupta, Yoon-Koo Kang, Patrick Schöffski, Jochen Schuette, Denis Soulières, Jean-Yves Blay, David Goldstein, Kolette Fly, Xin Huang, Massimo Corsaro, Maria Jose Lechuga, Jean-Francois Martini, Michael C. Heinrich

**Affiliations:** Department of Interdisciplinary Oncology, HELIOS Klinikum Berlin-Buch, Schwanebecker Chaussee 50, 13125 Berlin, Germany; Ludwig Center at Harvard and Dana-Farber Cancer Institute, Boston, MA USA; Leiden University Medical Center, Leiden, The Netherlands; Maria Sklodowska-Curie Memorial Cancer Center and Institute of Oncology, Warsaw, Poland; Seoul National University Hospital, Cancer Research Institute, Seoul National University College of Medicine, Seoul, South Korea; Tata Memorial Centre, Mumbai, India; Asan Medical Center, University of Ulsan College of Medicine, Seoul, South Korea; University Hospitals Leuven, Leuven Cancer Institute, and Laboratory of Experimental Oncology, KU Leuven, Leuven, Belgium; Hämatoonkologische Schwerpunktpraxis, Düsseldorf, Germany; Centre Hospitalier de l’Université de Montreal, Montreal, QC Canada; Centre Léon Bérard, Université Claude Bernard, Lyon, France; Prince of Wales Hospital, Sydney, Australia; Pfizer Oncology, Groton, CT USA; Pfizer Oncology, La Jolla, CA USA; Pfizer Oncology, Milan, Italy; VA Portland Health Care System and Oregon Health & Science University, Portland, OR USA

**Keywords:** Sunitinib, Imatinib, GIST, *KIT*, *KIT* mutation, Imatinib-resistant GIST, Overall survival, Progression-free survival

## Abstract

**Background:**

Several small studies indicated that the genotype of *KIT* or platelet-derived growth factor receptor-α (*PDGFRA*) contributes in part to the level of clinical effectiveness of sunitinib in gastrointestinal stromal tumor (GIST) patients. This study aimed to correlate *KIT* and *PDGFRA* mutational status with clinical outcome metrics (progression-free survival [PFS], overall survival [OS], objective response rate [ORR]) in a larger international patient population.

**Methods:**

This is a non-interventional, retrospective analysis in patients with imatinib-resistant or intolerant GIST who were treated in a worldwide, open-label treatment-use study (Study 1036; NCT00094029) in which sunitinib was administered at a starting dose of 50 mg/day on a 4-week-on, 2-week-off schedule. Molecular status was obtained in local laboratories with tumor samples obtained either pre-imatinib, post-imatinib/pre-sunitinib, or post-sunitinib treatment, and all available data were used in the analyses regardless of collection time. The primary analysis compared PFS in patients with primary *KIT* exon 11 versus exon 9 mutations (using a 2-sided log-rank test) and secondary analyses compared OS (using the same test) and ORR (using a 2-sided Pearson χ^2^ test) in the same molecular subgroups.

**Results:**

Of the 1124 sunitinib-treated patients in the treatment-use study, 230 (20 %) were included in this analysis, and baseline characteristics were similar between the two study populations. Median PFS was 7.1 months. A significantly better PFS was observed in patients with a primary mutation in *KIT* exon 9 (*n* = 42) compared to those with a primary mutation in exon 11 (*n* = 143; hazard ratio = 0.59; 95 % confidence interval, 0.39–0.89; *P* = 0.011), with median PFS times of 12.3 and 7.0 months, respectively. Similarly, longer OS and higher ORR were observed in patients with a primary *KIT* mutation in exon 9 versus exon 11. The data available were limited to investigate the effects of additional *KIT* or *PDGFRA* mutations on the efficacy of sunitinib treatment.

**Conclusions:**

This large retrospective analysis confirms the prognostic significance of *KIT* mutation status in patients with GIST. This analysis also confirms the effectiveness of sunitinib as a post-imatinib therapy, regardless of mutational status.

**Trial registration:**

NCT01459757.

**Electronic supplementary material:**

The online version of this article (doi:10.1186/s12885-016-2051-5) contains supplementary material, which is available to authorized users.

## Background

Gastrointestinal stromal tumors (GIST) comprise the most common primary mesenchymal malignancies of the gastrointestinal tract, and approximately 95 % of these tumors express the cell-surface transmembrane receptor KIT that has tyrosine kinase activity [1]. Constitutive activation of KIT occurs in approximately 80–85 % of cases through mutations at various sites in the transcribed sites of the *KIT* proto-oncogene [1]. This is one of the earliest cellular events responsible for the oncogenic transformation of GIST cells and is a key driver of the disease pathogenesis [2, 3]. Mutations occur most commonly in exon 11 (juxtamembrane domain), followed in frequency of incidence by exon 9 (extracellular domain) [1]. Activating mutations also occur in the *PDGFRA* gene (encoding the receptor tyrosine kinase platelet-derived growth factor receptor [PDGFR]-α) in approximately 5–7 % of GIST cases. These often occur mutually exclusively to *KIT* mutations, highlighting their important role in the pathogenesis of GIST [4]. Finally, there is a subset of 12–15 % of GIST cases which lack mutations in *KIT* and *PDGFRA* but which often harbor genomic or epigenetic aberrations in subunits of the succinate dehydrogenase (SDH) complex [5].

Imatinib is a relatively selective small molecule inhibitor of a limited number of tyrosine kinases—including KIT, PDGFRA, and the intracellular ABL kinase—that has helped to transform the management of GIST. It was approved for the treatment of metastatic or unresectable GIST in the USA in 2002, following a successful phase II trial and follow-up period [6, 7]. However, the clinical benefits observed in GIST patients with imatinib vary according to *KIT* and *PDGFRA* genotype. For example, patients with *KIT* exon 11-mutant GIST have a greater objective response rate (ORR) and longer median progression-free survival (PFS) with front-line imatinib treatment than GIST patients with *KIT* exon 9-mutant or *KIT/PDGFRA* “wild-type” (non-mutant) genotypes [8, 9]. Furthermore, the majority of patients with advanced GIST ultimately develop resistance to imatinib, which can either occur rapidly within 6 months of initiating therapy (primary resistance), or can appear with delay after 1 to more than 10 years on imatinib therapy. This delayed resistance usually occurs due to acquisition of secondary mutations in *KIT* or *PDGFRA* [10]. In the case of imatinib-resistant *KIT*-mutant GIST, these mutations cluster in the ATP-binding pocket (encoded by exons 13 and 14), and the activation loop (encoded by exons 17 and 18) of the kinase domain, and occur almost exclusively in the same gene and allele as the primary oncogenic driver mutation [10–13].

Sunitinib is a multi-targeted oral inhibitor of KIT, PDGFRs, vascular endothelial growth factor (VEGF) receptors (VEGFRs), and several other receptor tyrosine kinases [14–17]. It has shown clinically meaningful efficacy in phase I–III trials in imatinib-resistant or -intolerant patients with advanced GIST [18–21], and continues to be used worldwide after imatinib in this patient setting. However, as is the case with imatinib, the clinical effectiveness of sunitinib is influenced by mutations in the *KIT* and *PDGFRA* genes. Findings from several small studies indicate that endpoints such as PFS and overall survival (OS) are significantly longer for patients with primary (pre-imatinib) *KIT* exon 9 mutations compared with those with *KIT* exon 11 mutations in both Caucasian [22, 23] and, more recently, Asian populations [24]. Secondary mutation status may also have a prognostic role in sunitinib therapy success [22, 25–27], with data from small numbers of patients suggesting that mutation in exons 17 or 18 could confer some degree of resistance to the drug.

In the current study (Study 1199), we retrospectively examined correlations between clinical outcomes and *KIT*/*PDGFRA* mutational status in a subset of imatinib-resistant or -intolerant patients with GIST participating in a worldwide, open-label treatment-use study (Study 1036) [28].

## Methods

### Study design and patient selection

The current study (Study 1199; ClinicalTrials.gov identifier: NCT01459757) was designed as a non-interventional, retrospective analysis of *KIT/PDGFRA* mutation status data from an international open-label non-randomized, open-label treatment-use trial, Study 1036 (NCT00094029), which provided access to sunitinib to appropriate patients with GIST prior to availability of this agent in various countries around the world [28].

In the treatment-use study (Study 1036), 1131 patients were enrolled from 34 countries worldwide between September 2004 and December 2007, with 1124 patients receiving ≥1 dose of sunitinib (intent-to-treat [ITT] population). Key eligibility criteria included: age ≥18 years (however, protocol amendments also allowed younger patients to enroll), histologically confirmed metastatic and/or unresectable GIST not amenable to standard therapy, failed prior treatment with imatinib (indicated by disease progression or intolerance), potential to derive clinical benefit from sunitinib treatment, and resolution of all acute toxic effects of any prior therapy/surgery to grade ≤1. Sunitinib was administered at a starting dose of 50 mg/day on a 4-week-on, 2-week-off schedule (alternative dosing schedules were permitted following a protocol amendment in May 2006, which allowed patients to switch to 37.5 mg on a continuous daily dosing schedule). Treatment continued for as long as it was deemed to be clinically beneficial, as judged by the investigator. Tumor responses were assessed radiologically.

To be included in this retrospective sub-study (Study 1199), patients must have taken ≥1 dose of sunitinib in Study 1036, and have given consent for inclusion in the retrospective analysis (either personally or via the institutional review board/ethics committees if expired or lost to follow-up). Selection of participants was based upon the willingness of individual clinical study centers to participate, the availability of tumor mutational analysis data or retrievable tumor specimens for mutational analysis, and on the consent of patients. Additional outcomes data (PFS, OS, and ORR) from after the cutoff date of Study 1036 (July 2008), or not previously collected in Study 1036, were collated for analysis, where available and once the appropriate consent was obtained.

This study was conducted in accordance with the Declaration of Helsinki and Good Clinical Practice guidelines, and the protocol approved by the relevant institutional review board/independent ethics committees (see the Additional file 1 for a full list of the participating sites).

### Study objectives

The primary objective of this retrospective study was to correlate GIST genotype (specifically the *KIT* genotype within tumor cells) with clinical outcome (PFS and OS) in imatinib-resistant or intolerant patients with GIST treated with sunitinib. The secondary objective was to confirm the clinical efficacy of sunitinib therapy in imatinib-resistant or intolerant patients with advanced GIST.

### Assessments and analyses

#### Mutational status

Tumor tissue for mutational status assessment was obtained at any time point on one or more occasion (pre-imatinib, post-imatinib/pre-sunitinib, or post-sunitinib treatment). Mutational status of the relevant kinase targets was determined by local laboratory analyses. *KIT* mutation locations were noted where available (e.g. exon 9, exon 11, and exon 13), together with the location of *PDGFRA* mutations (e.g. exon 12, exon 18). If no mutations were found, patients were classified as either “*KIT* and *PDGFRA* wild-type”, when all key exons (*KIT* exons 9, 11, 13, and 17; *PDGFRA* exons 12 and 18) were assessed and no mutations were found, or “mutation-absent” if no mutations were found, but only a subset of the key exons were assessed. Available mutational data were used in all analyses, regardless of the time of collection.

#### Efficacy analysis

The definition of PFS was the time from date of enrollment in Study 1036 to first progression of disease (PD) or death for any reason in the absence of documented PD (up to last dose date + 28 days), whichever occurred first. OS was defined as the time from date of enrollment in Study 1036 to date of death due to any cause.

The definition of ORR was the percent of patients achieving a confirmed complete response (CR) or partial response (PR) in Study 1036, according to the Response Evaluation Criteria in Solid Tumors (RECIST) version 1.0 [29]. Confirmed responses were those that persisted on repeat imaging at least 4 weeks after the initial documentation of response. Patients who did not have on-study radiographic tumor re-evaluation, who received anti-tumor treatment other than the study medication prior to reaching a CR or PR, or who died, progressed, or dropped out for any reason prior to reaching a CR or PR were counted as non-responders in the assessment of ORR.

#### Adverse events

Adverse events (AEs) were assessed until ≤28 days after the last dose of sunitinib and graded using National Cancer Institute Common Terminology Criteria for Adverse Events version 3.0.

### Statistical analysis

Analyses were performed for all patients who received ≥1 dose of sunitinib and from whom consent was obtained (full analysis set). The sample size for this study was calculated using PFS assumptions that were based on the results of a phase II sunitinib study [22].

In the study, PFS (in months) was calculated as: (first event date − enrollment date +1)/30.4. Additionally, OS (in months) was calculated as: (date of death − enrollment date +1)/30.4. For patients still alive at the time of the analysis or without confirmation of death, the OS time was censored on the last date they were known to be alive. Patients lacking data beyond enrollment had their OS times censored at enrollment with a duration of 1 day. PFS and OS were summarized using Kaplan–Meier methods.

The median PFS or OS time and corresponding 2-sided 95 % confidence intervals (CIs) were also calculated based on *KIT* mutational status. A 2-sided log-rank test was used to compare PFS (primary analysis) or OS between patients with primary *KIT* exon 9 and exon 11 mutations, with a significance level of 0.05. The hazard ratio (HR) and its 95 % CIs were estimated. Cox proportional hazard models were used to evaluate whether other baseline characteristics, including age and performance status, could also influence PFS and OS over and above primary mutational status.

The number and percent of patients achieving an objective response (CR or PR) were summarized along with the corresponding exact 2-sided 95 % CI based on *KIT* mutational status. In this regard, ORR by primary *KIT* mutational status (exon 11 versus exon 9) was compared using a 2-sided Pearson χ^2^ test to significance level 0.05, with corresponding 2-sided 95 % CIs estimated using the exact method based on the F-distribution.

## Results

### Study disposition and baseline characteristics

Of the 1124 patients in the treatment-use, Study 1036, who received ≥1 dose of sunitinib, 230 (20 %) were included in this retrospective analysis (Study 1199), based upon clinical center participation, patient consent, and genotype data availability. Despite the non-random selection of patients in Study 1199, the baseline demographic and clinical characteristics of these patients were representative of the larger population in Study 1036 (Table 1). Both studies had a similar median age (60 years in this study; 59 years in Study 1036). Additionally, the studies had similar distributions of patients by sex (60 % male in both studies), race (80 % and 76 % white in this study and Study 1036, respectively), and baseline Eastern Cooperative Oncology Group (ECOG) performance status (87 % and 84 % ECOG 0 or 1 in this and Study 1036, respectively). The respective median times since original diagnosis were 186 and 171 weeks in studies 1199 and 1036, and a 600–800 mg maximum prior imatinib dose was the most frequent in both studies (43 % in Study 1199 and 47 % in Study 1036). The outcome of PD within and beyond 6 months of the start of prior imatinib therapy was seen in 13 % and 79 % of patients in Study 1199, respectively, and 14 % and 77 % of patients in Study 1036, respectively. A CR or PR to prior imatinib treatment was observed in 39 % of patients in Study 1199 and 36 % of patients in Study 1036. Overall, 8 % and 9 % of patients were intolerant to prior imatinib therapy in studies 1199 and 1036, respectively. Additional treatment history (i.e. other than imatinib) of patients in Study 1199 and Study 1036 was also comparable: systemic therapy other than imatinib was received by 21 % and 20 % of patients, respectively; previous surgery was performed in 99 % and 98 % of patients, respectively; and previous radiotherapy was received by 7 % of patients in both studies.

**Table 1:** Baseline clinical characteristics in Studies 1199 and 1036

Characteristic	Study 1199 (*N* = 230)	Study 1036 (*N* = 1124)
Age, median (range), years	60 (11−83)	59 (10−92)
Male sex, n (%)	139 (60)	672 (60)
ECOG performance status,^a^ n (%)		
0	87 (38)	420 (37)
1	114 (50)	521 (46)
2	24 (10)	135 (12)
3	3 (1)	33 (3)
4	0	5 (<1)
Time since original diagnosis, median (range), weeks	186 (12–773)	171 (3−1584)
Maximum prior imatinib dose,^b^ n (%)		
≤ 400 mg	71 (31)	353 (31)
> 400–600 mg	58 (25)	212 (19)
> 600–800 mg	99 (43)	532 (47)
> 800 mg	2 (1)	24 (2)
Outcome with prior imatinib therapy,^c^ n (%)		
PD within 6 months of start	30 (13)	153 (14)
PD beyond 6 months of start	181 (79)	871 (77)
Intolerance	19 (8)	99 (9)
Best response to prior imatinib,^d^ n (%)		
CR	8 (3)	56 (5)
PR	82 (36)	353 (31)
Stable disease	93 (40)	391 (35)
PD	39 (17)	288 (26)
Not applicable	7 (3)	31 (3)

Study 1199: full analysis population; Study 1036: ITT population

CR complete response, ECOG Eastern Cooperative Oncology Group, ITT intent-to-treat, PD progressive disease, PR partial response

^a^Data missing: Study 1199, *n* = 2; Study 1036, *n* = 10

^b^Data missing: Study 1036, *n* = 3

^c^Data missing: Study 1036, *n* = 1

^d^Data missing: Study 1199, *n* = 1; Study 1036, *n* = 5

### Mutational status *of KIT* and *PDGFRA* target genes

Table 2 and Additional file 1: Table S1 show *KIT* and *PDGFRA* mutational status data for the full analysis set of Study 1199. For the analysis of primary *KIT* mutational status, samples were collected from 148 patients, pre-imatinib treatment, from 68 patients, post-imatinib/pre-sunitinib treatment, and from 24 patients, post-sunitinib treatment (see Additional file 1: Table S2 for the distribution of primary *KIT* exon 9 and exon 11 mutations according to sampling time point). Overall, 86 % of patients had any primary *KIT* mutation. The most frequent *KIT* primary mutations occurred in exon 11 (62 %) and exon 9 (18 %), and 4 % of patients were classified as wild-type (no mutation in *KIT* exons 9, 11, 13, and 17) (Table 2). Secondary *KIT* mutations occurred in 11 % of patients and were observed most frequently in exons 13 and 17 (in 5 % of patients each). Third *KIT* mutations were only observed in two patients, although this information was classified as “absent” or “missing” in 99 % of patients (Additional file 1: Table S1).

**Table 2:** Primary *KIT* and *PDGFRA* mutational status in Study 1199

Mutational status	Study 1199 (*N* = 230) n (%)
*KIT* primary mutation	
Any	197 (86)
Exon 9	42 (18)
Exon 11	143 (62)
Exon 13	5 (2)
Exon 17	6 (3)
Other	1 (<1)
Wild-type	9 (4)
Absent^a^	23 (10)
Missing^b^	1 (<1)
*PDGFRA* primary mutation	
Any	18 (8)^c^
Exon 10	1 (<1)
Exon 12	1 (<1)
Exon 18	5 (2)
Other	11 (5)^c^
Absent^a^	97 (42)
Missing^b^	115 (50)

PDGFRA, platelet-derived growth factor receptor-α

^a^Mutational status was classified as “absent” if no mutations were found but only a subset of the key exons were assessed

^b^Mutational status was classified as “missing” if no assessments were performed

^c^6 patients (3%) with tumor genotypes classified as "other" were wild-type for *PDGFRA* mutations status

Demographic and baseline clinical characteristics were generally similar across all primary *KIT* mutational status groups and the study population as a whole (Table 3). However, as expected, when considering prior imatinib therapy, the proportion of patients with PD within 6 months of treatment initiation was significantly lower among those with exon 11 mutations (3 %) when compared with other mutational groups (17–43 %). Conversely, PD seen beyond 6 months of imatinib treatment initiation was observed at a higher frequency among those with exon 11 mutations (92 %) compared with those in other mutational groups (44–62 %). Similarly, patients with exon 11 mutations displayed a better response (CR + PR) to prior imatinib treatment when compared with those with exon 9 mutations (52 % and 21 %, respectively).

**Table 3:** Baseline clinical characteristics based on primary *KIT* mutational status^a^

Characteristic	Absent (*n* = 23)	Exon 9 (*n* = 42)	Exon 11 (*n* = 143)	Exon 13 (*n* = 5)	Exon 17 (*n* = 6)	Wild-type (*n* = 9)
Age, median (range), years	54 (11–67)	58 (26–79)	61 (27–83)	67 (31–75)	62 (44–79)	46 (33–64)
Male sex, n (%)	10 (43)	29 (69)	88 (62)	4 (80)	3 (50)	4 (44)
ECOG performance status,^b^ n (%)						
0	10 (43)	20 (48)	50 (35)	1 (20)	3 (50)	2 (22)
1	9 (39)	17 (40)	73 (51)	4 (80)	3 (50)	7 (78)
2	4 (17)	4 (10)	16 (11)	0	0	0
3	0	0	3 (2)	0	0	0
4	0	0	0	0	0	0
Time since original diagnosis, median (range), weeks	92 (12–310)	142 (22–702)	209 (16–680)	118 (33–236)	197 (21–545)	177 (30–773)
Maximum prior imatinib dose, n (%)						
≤ 400 mg	8 (35)	10 (24)	51 (36)	0	0	2 (22)
> 400–600 mg	7 (30)	8 (19)	37 (26)	4 (80)	2 (33)	0
> 600–800 mg	8 (35)	24 (57)	53 (37)	1 (20)	4 (67)	7 (78)
> 800 mg	0	0	2 (1)	0	0	0
Outcome with prior imatinib therapy,^c^ n (%)						
PD within 6 months of start	10 (43)	10 (24)	4 (3)	2 (40)	1 (17)	3 (33)
PD beyond 6 months of start	12 (52)	26 (62)	131 (92)	3 (60)	3 (50)	4 (44)
Intolerance	1 (4)	6 (14)	8 (6)	0	2 (33)	2 (22)
Best response to prior imatinib,^d^ n (%)						
CR	0	2 (5)	6 (4)	0	0	0
PR	3 (13)	7 (17)	68 (48)	0	3 (50)	1 (11)
Stable disease	9 (39)	22 (52)	51 (36)	2 (40)	2 (33)	6 (67)
PD	10 (43)	10 (24)	14 (10)	3 (60)	1 (17)	1 (11)
Not applicable	1 (4)	1 (2)	4 (3)	0	0	0

CR complete response, ECOG Eastern Cooperative Oncology Group, PD progressive disease, PR partial response

^a^Data not shown for other mutations (*n* = 1) or data missing (*n* = 1)

^b^Data not shown for ECOG performance status missing (exon 9, *n* = 1; exon 11, *n* = 1; all other groups, *n* = 0)

^c^No data missing

^d^Data not shown for response missing (wild-type, *n* = 1; all other groups *n* = 0)

Primary *PDGFRA* mutational status was missing for 50 % of patients, and primary *PDGFRA* mutations were observed in only 12 patients (5 %), most often in exon 18 (*n* = 5). No secondary *PDGFRA* mutations were observed.

### Sunitinib efficacy overall and by mutational status

It was possible to analyze the efficacy of sunitinib treatment in the context of patients with primary *KIT* exon 9 or exon 11 mutations. Unfortunately, the available data were too limited to investigate the effects of additional *KIT* mutations, or the effects of *PDGFRA* mutations, on efficacy outcomes following sunitinib treatment. Similarly, the effect of the wild-type genotype could not be investigated due to the limited number of patients in this category (*n* = 9).

### Correlation between *KIT* mutations and progression-free survival with sunitinib

Overall, median PFS in the Study 1199 patient population was representative of Study 1036 following sunitinib treatment: 7.1 months (95 % CI: 6.4–8.1 months) and 7.6 months (95 % CI: 6.8–8.1 months), respectively.

**Fig. 1:**
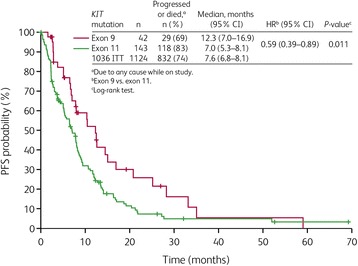
Progression-free survival (PFS) by primary *KIT* mutational status in Study 1199. CI, confidence interval; HR, hazard ratio; ITT, intent-to-treat

With sunitinib treatment, patients with a primary *KIT* mutation in exon 9 displayed a significantly better PFS compared with those with a primary mutation in exon 11 (HR = 0.59; 95 % CI: 0.39–0.89; *P* = 0.011; Fig. 1), with median PFS times of 12.3 and 7.0 months, respectively. In addition, the proportion of patients who progressed or died at the time of the analysis was lower (69 %) for patients with primary *KIT* exon 9 mutations compared with those with primary *KIT* exon 11 mutations (83 %). The Cox proportional hazards analysis of PFS revealed that neither age (<59 versus ≥59 years; HR = 1.02; 95 % CI: 0.73–1.41) nor baseline ECOG performance status (<2 versus ≥2; HR = 0.74; 95 % CI: 0.45–1.20) had a significant additional effect on PFS.

### Correlation between *KIT* mutations and overall survival with sunitinib

Across the whole study population, OS was similar between studies 1199 and 1036, with a median of 19.3 months (95 % CI: 15.9–22.5 months) and 16.6 months (95 % CI: 14.9–18.0 months) [28], respectively.

**Fig. 2:**
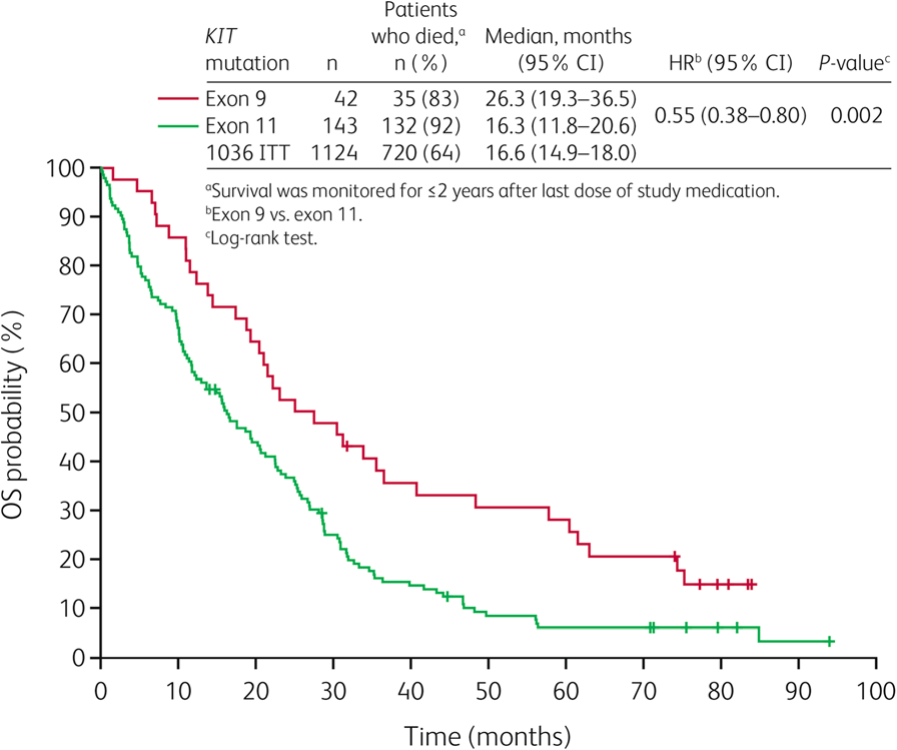
Overall survival (OS) in Study 1199 by primary *KIT* mutational status. CI, confidence interval; HR, hazard ratio; ITT, intent-to-treat

As with PFS, patients with a primary *KIT* mutation in exon 9 displayed a significantly better OS when compared with those with a primary *KIT* mutation in exon 11 (HR = 0.55; 95 % CI: 0.38–0.80; *P* = 0.002; Fig. 2), with median OS times of 26.3 and 16.3 months, respectively. Furthermore, the proportion of patients who had progressed or died at the time of the analysis tended to be lower (83 %) for patients with primary exon 9 mutations than for those with primary exon 11 mutations (92 %). The Cox proportional hazards analysis of OS revealed ECOG performance status (<2 versus ≥2) had a significant effect on OS in addition to mutational status (HR for ECOG = 0.58; 95 % CI: 0.37–0.91), but age (<59 versus ≥59 years) did not (HR = 1.01; 95 % CI: 0.74–1.37).

### Correlation between *KIT* mutations and overall objective response rates with sunitinib

The overall ORR was similar between the two study populations: 8 % (95 % CI: 5–12) and 8 % (95 % CI: 6–10) for studies 1199 and 1036 [28], respectively.

In the present analysis, patients with a primary *KIT* exon 9 mutation had a significantly higher ORR than those with a primary *KIT* exon 11 mutation (19 % versus 6 %; *P* = 0.012; Table 4). Of the eight patients with primary *KIT* mutations in exon 9 (*n* = 42) who achieved an objective response (CR + PR), two achieved a CR (5 % overall; 25 % of the objective response group). All nine of the responses observed in patients with primary KIT exon 11 mutations (*n* = 143) were PRs.

**Table 4:** Best objective tumor response (investigator assessment) in Study 1199 by primary *KIT* mutational status, and in the overall ITT population of Study 1036

Response parameter	Study 1199		1036 ITT (*N =* 1124)
	Exon 9 (*n* = 42)	Exon 11 (*n* = 143)	
Best confirmed tumor response,^a^ n (%)			
CR	2 (5)	0	10 (1)
PR	6 (14)	9 (6)	78 (7)
Stable disease	29 (69)	86 (60)	639 (57)
PD	4 (10)	31 (22)	237 (21)
Not evaluable	0	1 (1)	2 (<1)
Missing	1 (2)	16 (11)	158 (14)
Confirmed objective responses,^b^ n (%)	8 (19)	9 (6)	88 (8)
95 % exact CI, %	9−34	3−12	6−10
Difference in ORR: exon 9 vs. exon 11, %	13		NA
95 % CI	<1−25		NA
*P*-value (two-sided Pearson χ^2^)	0.012		NA

CI confidence interval, CR complete response, ITT intent-to-treat, NA not applicable, PD progressive disease, PR partial response

^a^Tumor assessment data obtained ≤28 days after last dose of study drug

^b^CR  + PR

### Adverse events

In general, the safety profile observed with sunitinib in the 1199 study population was similar to the profile seen in Study 1036. Treatment-related AEs were observed in 93 % and 92 % of patients in studies 1199 and 1036, respectively. Serious treatment-related AEs occurred in 21 % and 22 % of patients, respectively. The proportion of patients experiencing AEs leading to treatment discontinuation was 30 % for both studies.

## Discussion

Although effective in the vast majority of patients, the eventual evolution of resistance to imatinib is common in patients with GIST, with resistance observed in more than 80 % of evaluable patients during long-term follow-up in a phase III trial [30]. Sunitinib, a multi-targeted inhibitor of KIT and other receptor tyrosine kinases, is an important therapy for patients with GIST who become resistant to, or are intolerant of, imatinib. This non-interventional retrospective analysis (Study 1199) provided further evidence that imatinib-resistant or -intolerant patients with GIST experience clinical benefit from sunitinib treatment, regardless of the mutational status of their tumor. No mutational subsets of patients were found in the current study in which the drug was inactive, although this retrospective analysis had limited ability to assess very rare mutational subtypes or impact of various mutations in *PDGFRA*.

Clinical benefit associated with sunitinib-induced control of GIST is thought to be influenced by *KIT* mutational status [22, 24–26, 31], and identifying those patients who are most likely to benefit from sunitinib treatment is desirable for both patients and clinicians. The current study aimed to correlate *KIT* mutational status data with clinical outcome in sunitinib-treated patients with imatinib-resistant or -intolerant GIST. The primary analysis revealed that individuals with a primary *KIT* exon 9 mutation in their tumor achieved better clinical outcomes during treatment with sunitinib than those with a primary *KIT* exon 11 mutation, across all three efficacy measures (PFS, OS, and ORR). Patients with *KIT* exon 9 mutations experienced a 41 % reduction in risk of progression, a 45 % reduction in the risk of death, and ORR that was three times higher, compared to those with primary *KIT* mutations in exon 11.

The observation that patients with GIST with a primary *KIT* mutation in exon 9 present with better outcomes following sunitinib treatment compared with those with a primary *KIT* mutation in exon 11 is consistent with previous studies in both Caucasian [22, 23] and Asian [24] populations. However, these studies involved relatively small patient sets of less than 100 subjects, resulting in low numbers of individuals within the different mutation subgroups. In contrast, the present study utilized data from a much larger cohort of 230 patients, including 42 harboring *KIT* exon 9 mutations and 143 with exon 11 mutations. The favorable outcomes in GIST with primary *KIT* mutations in exon 9 treated with sunitinib are consistent with in-vitro data, demonstrating greater potency of sunitinib over imatinib against exon 9 mutant KIT, and similar potency of each drug against exon 11 mutant KIT [22]. This observation could be due to the differential effect that mutation within each site has upon KIT receptor structure [32, 33].

Despite the non-randomized patient selection for this retrospective analysis, the baseline demographic and disease characteristics were similar to those observed in the parent Study 1036, which enrolled over 1000 patients with GIST from centers worldwide [28]. In addition, differences in dosing were unlikely to have impacted the findings. Previously reported post-hoc analyses of Study 1036 found that patients who received sunitinib on an alternative dosing schedule versus those who received only the initial dosing schedule had prolonged treatment, which may have led to improved outcomes, including prolonged TTP and OS [28]. However, among the 230 patients in the current study, 108 (47 %) had a dose reduction, similar to the number of patients in the parent study (43 %). Furthermore, the numbers of patients with dose reductions in the exon 9 and exon 11 subgroups in this study were comparable (both 48 %). Therefore, the results from the current study are likely to be broadly representative of the usual population that clinicians will see in everyday practice. In this respect, it is noteworthy that the distribution of primary KIT and PDGFRA mutations in our study was consistent with previous studies among patients with GIST [1, 8, 22]. Overall, 86 % of participants in our analysis had a primary mutation in *KIT* and 5 % had a primary mutation in *PDGFRA.*

Other secondary mutations may also influence response to sunitinib [22, 25–27]. Data in small numbers of patients indicate that mutations in exons 17 and 18 may confer some degree of resistance to the drug. Unfortunately, information on secondary and tertiary mutation status of the patients in this study was not analyzable due to the limited availability of data. This was because, given the retrospective nature of this analysis, in some cases, only one biopsy was taken for each patient and also because biopsy collection timings varied between patients. Some biopsies were collected before beginning first-line treatment with imatinib (pre-imatinib, *n* = 148), some during or after completion of first-line treatment with imatinib, but before beginning treatment with sunitinib (post-imatinib/pre-sunitinib; *n* = 68), and some after the beginning or after completion of treatment with sunitinib (post-sunitinib; *n* = 24). This represents a limitation of this study. It should also be noted that only a subset of patients in the treatment-use study (Study 1036) were included in this correlation study; thus, this selected group of patients may not be fully representative of the pool of patients with secondary mutations. Finally, it should be noted that different mutational subtypes of *KIT* exon 9 and 11 may have a differential impact on treatment outcome (e.g. gastric GISTs with exon 11 deletions are more aggressive than those with substitutions) [34]; however, due to the limited number of patients, this level of analysis could not be performed in the current study.

Combined with the existing evidence, our data suggest that obtaining information on *KIT* mutations from patients before the start of treatment would allow clinicians to predict who are most likely to experience resistance to primary imatinib therapy, to evaluate which patients would benefit the most from sunitinib therapy, and also to aid in our understanding of why particular patients respond better than others. The data also support stratification by mutational status in future trials comparing sunitinib and novel agents. However, extensive intra- and inter-lesional heterogeneity of resistance mutations in patients with clinically progressing GIST is apparent, with up to five different secondary mutations observed in different metastases and up to two in the same metastasis in one study [35]. As a result of this, the information that can be generated from mutational analysis of a discrete, single tumor biopsy at the time of progression may confound subsequent treatment decisions. In the future, a meta-analysis of studies will be worthwhile to study the influence of rare mutations on outcomes in patients treated with sunitinib, and next-generation sequencing may provide more information on predominant and minor mutations that influence the efficacy of sunitinib and other agents. In addition, it must be remembered that mutational status is not the only prognostic factor that influences the clinical outcome of patients with GIST on receptor tyrosine kinase therapy, with initial low tumor volume, female gender, and CD34 positivity predicting higher PFS in a recent study considering patients treated with imatinib [36]. There is also evidence that exon 9-mutated GIST metastasizes significantly more often to the peritoneum than to the liver and that exon 9 mutations per se may not have prognostic relevance [37]; however, we do not have the level of data required to test a possible correlation of primary *KIT* mutation with metastasis status and location. Another important element of the multiple mechanisms of action of sunitinib as it pertains to GIST tumor biology is the complexity of the angiogenesis process. Expression of VEGF (a highly pro-angiogenic ligand of VEGFR2, which is another target of sunitinib but not of imatinib) has been shown to be higher in wild-type GISTs than in *KIT*-mutant GIST [38], and little is known about the angiogenic status at the time of progression on imatinib, which is likely to play a role in the mechanisms of resistance, as with many other targeted therapies. Finally, a recently reported study of theranostic biomarkers that identified potential therapies beyond tyrosine kinase inhibitors for GIST, including various cytotoxics and non-KIT/PDGFRA targeted therapies, underscores the heterogeneous nature of GIST [39].

## Conclusions

In summary, this large retrospective study provides a robust analysis of the influence of *KIT* mutational status on clinical outcomes with sunitinib in patients with advanced GIST following failure of imatinib due to resistance or intolerance. The study also confirms the effectiveness of sunitinib as a post-imatinib therapy in patients, regardless of the mutational status of their tumor. It also confirms differential activity in *KIT* exon 9 versus exon 11 patients and adds to the limited data available on sunitinib activity in patients with other GIST mutations or SDH-deficient (“*KIT/PDGFRA* wild-type”) tumors. These data should give clinicians increased confidence in the effectiveness of sunitinib in all of these particular GIST patient subsets.

### Availability of data and materials

The data supporting this manuscript are located at Pfizer and available for inspection upon request.

## Additional file

Additional file 1:
**Supplementary Methods and Results. **(DOC 64 kb)
