# Supplementary material for: Correlation of KIT and PDGFRA mutational status with clinical benefit in patients with gastrointestinal stromal tumor treated with sunitinib in a worldwide treatment-use trial
Source: BMC Cancer. 2016 Jan 15;16:22. doi: 10.1186/s12885-016-2051-5 (PMC4714485; doi:10.1186/s12885-016-2051-5)
Supplement: Additional file 1: — Supplementary Methods and Results. (DOC 64 kb) [file 12885_2016_2051_MOESM1_ESM.doc]

**Supplementary Material**

**Supplementary Methods**

This study was conducted in accordance with the Declaration of Helsinki and Good Clinical Practice guidelines, and the protocol approved by the relevant institutional review board/independent ethics committees at the following participating sites:

**Australia:** Ashford Cancer Centre Research, Kurralta Park; Peter MacCallum Cancer Institute, Department of Medical Oncology, East Melbourne; Prince of Wales Hospital, Oncology Day Center, Randwick; **Belgium:** UZ Gasthuisberg, Oncology, Leuven; **Canada:** Hopital Notre-dame du Centre Hospitalier, Universitaire de Montreal, Oncology Center, Montreal; **Denmark:** Herlev Hospital, Onkologisk afdeling, Herlev; **Finland:** Helsingin yliopistollinen keskussairaala/Syopatautien klinikka, Helsinki; **France:** Centre Leon Berard, Lyon; CHU La Timone, Service d'Oncologie Medicale, Marseille; Institut Bergonie, Bordeaux; **Germany:** Klinikum der Universitaet zu Koeln, Innere Medizin; Koeln; Schwerpunktpraxis fuer ambulante Tumortherapie, Haematologie und Onkologie, Duesseldorf; HELIOS Klinikum Berlin-Buch, Klinik fuer Interdisziplinaere Onkologie, Sarkomzentrum Berlin-Brandenburg, Berlin; **India:** Tata Memorial Hospital, Department of Medical Oncology, Mumbai; **Korea:** Seoul National University Hospital/Department of Internal Medicine, Seoul; Asan Medical Center, Department of Oncology, Seoul; **Netherlands:** Leids Universitair Medisch Centrum/ Klinische Oncologie, Leiden; **Poland:** Klinika Nowotworow Tkanek Miekkich, Kosci i Czerniakow, Centrum Onkologii-Instytut im. Marii Sklodowskiej-Curie, Warszawa; **Slovakia:** Narodny Onkologicky ustav, Bratislava; **Spain:** Hospital de la Santa Creu i Sant Pau, Servicio Oncologia Medica - Bloque A, Barcelona; Hospital Universitario Virgen Macarena, Servicio de Oncologia, Sevilla; **Switzerland:** Centre Hospitalier Universitaire Vaudois, Centre Pluridisciplinaire d'Oncologie, Lausanne; **UK:** Christie Hospital NHS Trust, Manchester; Department of Medicine, The Royal Marsden NHS Foundation Trust, London; **USA:** Dana-Farber Cancer Institute - Center for Sarcoma and Bone Oncology, Boston, MA; City of Hope - Department of Medical Oncology, Duarte, CA; Moffitt Cancer Center, Sarcoma Department, Tampa, FL; Masonic Cancer Center, Minneapolis, MN; Seattle Cancer Care Alliance, Seattle, WA; Oregon Health and Science University, Portland, OR; University of Florida College of Medicine, Gainesville, FL; Washington Hospital Center, Washington, DC.

**Supplementary Table S1 Secondary and tertiary *KIT* and *PDGFRA* mutational status in Study 1199**

| **Mutational status** | **Study 1199 (N = 230)**  **n (%)** |
| --- | --- |
| Second *KIT* mutation |  |
| Any | 26 (11) |
| Exon 11 | 1 (<1) |
| Exon 13 | 12 (5) |
| Exon 14 | 0 (0) |
| Exon 17 | 12 (5) |
| Exon 18 | 0 |
| Other | 1 (<1) |
| Wild-type | 0 |
| Absent^a^ | 75 (33) |
| Missing^b^ | 129 (56) |
| Third *KIT* mutation |  |
| Any | 2 (1) |
| Exon 13 | 1 (<1) |
| Other | 1 (<1) |
| Absent^a^ | 79 (34) |
| Missing^b^ | 149 (65) |
| Second *PDGFRA* mutation |  |
| Other | 0 |
| Absent^a^ | 68 (30) |
| Missing^b^ | 162 (70) |

PDGFRA, platelet-derived growth factor receptor-α.

^a^Mutational status was classified as “absent” if no mutations were found but only a subset of the key exons were assessed.

^b^Mutational status was classified as “missing” if no assessments were performed.

**Supplementary Table S2 Distribution of primary *KIT* exon 9 and exon 11 mutations according to sampling time point**

| **Sample time point** | **Exon 9** | **Exon 11** |
| --- | --- | --- |
| Pre-imatinib | 23 | 91 |
| Post-imatinib/pre-sunitinib | 14 | 45 |
| Post-sunitinib | 6 | 12 |
